# PhP.B Enhanced Adeno-Associated Virus Mediated-Expression Following Systemic Delivery or Direct Brain Administration

**DOI:** 10.3389/fbioe.2021.679483

**Published:** 2021-08-03

**Authors:** Kimberly L. Pietersz, Francois Du Plessis, Stephan M. Pouw, Jolanda M. Liefhebber, Sander J. van Deventer, Gerard J. M. Martens, Pavlina S. Konstantinova, Bas Blits

**Affiliations:** ^1^Department of Research & Development, uniQure Biopharma B.V., Amsterdam, Netherlands; ^2^Department of Molecular Animal Physiology, Faculty of Science, Centre for Neuroscience, Donders Institute for Brain, Cognition and Behavior, Radboud University, Nijmegen, Netherlands; ^3^Department of Gastroenterology and Hepatology, Leiden University Medical Center, Leiden, Netherlands

**Keywords:** aav, intrastriatal, intravenous, CNS, PhP.B

## Abstract

Of the adeno-associated viruses (AAVs), AAV9 is known for its capability to cross the blood–brain barrier (BBB) and can, therefore, be used as a noninvasive method to target the central nervous system. Furthermore, the addition of the peptide PhP.B to AAV9 increases its transduction across the BBB by 40-fold. Another neurotropic serotype, AAV5, has been shown as a gene therapeutic delivery vehicle to ameliorate several neurodegenerative diseases in preclinical models, but its administration requires invasive surgery. In this study, AAV9-PhP.B and AAV5-PhP.B were designed and produced in an insect cell–based system. To AAV9, the PhP.B peptide TLAVPFK was added, whereas in AAV5-PhP.B (AQTLAVPFKAQAQ), with AQ-AQAQ sequences used to swap with the corresponding sequence of AAV5. The addition of PhP.B to AAV5 did not affect its capacity to cross the mouse BBB, while increased transduction of liver tissue was observed. Then, intravenous (IV) and intrastriatal (IStr) delivery of AAV9-PhP.B and AAV5 were compared. For AAV9-PhP.B, similar transduction and expression levels were achieved in the striatum and cortex, irrespective of the delivery method used. IStr administration of AAV5 resulted in significantly higher amounts of vector DNA and therapeutic miRNA in the target regions such as striatum and cortex when compared with an IV administration of AAV9-PhP.B. These results illustrate the challenge in developing a vector that can be delivered noninvasively while achieving a transduction level similar to that of direct administration of AAV5. Thus, for therapeutic miRNA delivery with high local expression requirements, intraparenchymal delivery of AAV5 is preferred, whereas a humanized AAV9-PhP.B may be useful when widespread brain (and peripheral) transduction is needed.

## Graphical Abstract

Comparison of AAV5 vs. AAV9-PhP.B delivered intravenous or intrastriatal. Irrespective of the two delivery methods, similar transduction in the cortex and striatum is achieved with AAV9-PhP.B. In contrast, AAV5—when injected directly into the striatum—obtains high transduction levels in the cortex and striatum.

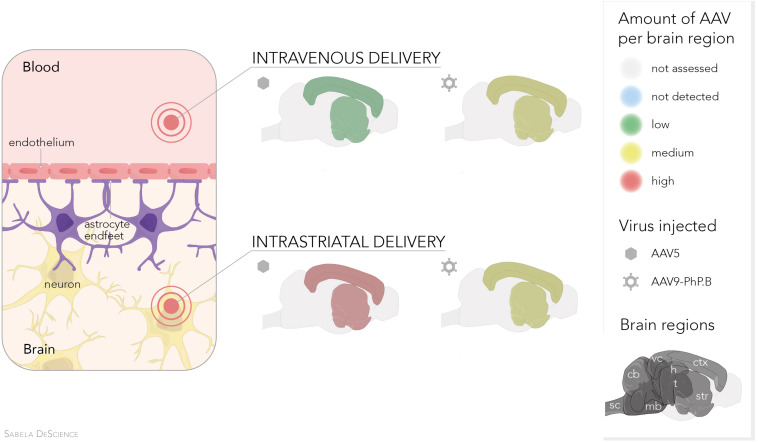

## Introduction

Neurodegenerative diseases are a heterogeneous group of multisystem disorders affecting the central nervous system (CNS), and treatment options for those are limited. An important and challenging aspect of the treatment of these diseases is efficient drug target delivery. Viral vectors harboring therapeutic nucleic acid molecules have to be delivered to the CNS to accomplish their therapeutic action at the desired site. The delivery of viral vectors is a part of molecular neurosurgery, thereby treating disease on a molecular level. Various disease-modifying therapies are in the (pre)clinical stage for neurodegenerative diseases based on adeno-associated virus (AAV) as the delivery tool of nucleic acids (Chen et al., 2020; Mijanović et al., 2020).

One of the examples of such a preclinical program is for the treatment of Huntington’s disease (HD). Intrastriatal (IStr) delivery of AAV carrying a huntingtin silencing miRNA resulted in a successful reduction in huntingtin mRNA and protein in the brains of mouse and minipig HD models (Evers et al., 2018; Spronck et al., 2019). The produced miRNA lowered cytoplasmic and nuclear gene expression and showed therapeutic spread through extracellular vesicles without off-target effects (Keskin et al., 2019). This miRNA technology has also been used as silencing strategies for Spinocerebellar Ataxia Type 3 (Martier et al., 2019b) and Amyotrophic Lateral Sclerosis (ALS) (Martier et al., 2019a).

The direct IStr administration of AAV has been used to supply the brain locally with the therapeutic doses of the miRNA (Evers et al., 2018; Spronck et al., 2019). However, this invasive intervention requires a well-equipped and trained neurosurgical team. Intravenous (IV) administration would be an attractive alternative since it is less invasive. Nevertheless, an AAV capsid that can cross the blood–brain barrier (BBB) is required when targeting the CNS by IV administration. AAV9 has been shown to have this capability, especially in neonates (Foust et al., 2008). The number of vector copies needed to reach therapeutic doses in the brain may lead to elevated aminotransferase levels, an indicator of liver damage (Mendell et al., 2017). For the probable cause of systemic adverse events, it is currently challenging to use IV administration of AAV9 for neurodegenerative diseases in adults.

Various attempts have been undertaken to improve the capability and efficiency of AAV to cross the BBB. A variant of AAV9, named AAV9-PhP.B, was reported to enhance brain tissue transduction, such as the cortex, by 40-fold compared with its AAV9 ancestor (Deverman et al., 2016). The enhancement of AAV9 by PhP.B appears to occur only in rodents (Hordeaux et al., 2018), with a preference for the C57Bl/6 strain, the strain in which PhP.B was developed and tested (Hordeaux et al., 2018). Studies in nonhuman primates (Liguore et al., 2019) and marmosets (Matsuzaki et al., 2018) have not shown any increase in the transduction levels of AAV9-PhP.B when compared to AAV9. PhP.B binds to the Ly6a receptor, a GPI (glycophosphatidylinositol)-anchored surface protein highly expressed in the microvascular endothelial cells of C57Bl/6 mice (Hordeaux et al., 2019; Huang et al., 2019). Unfortunately, Ly6a is absent in endothelial cells of primates (Hordeaux et al., 2019), which explains why no increase is observed in BBB crossing compared with AAV9. These results limit the use of PhP.B-containing vectors in humans. However, AAV9-PhP.B can still be used as a benchmark for preclinical studies in C57Bl/6 mice. The knowledge gained from these studies could be used to rationally develop novel vectors that cross the BBB.

PhP.B is constructed by inserting a 7-mer peptide at position I-588. The insertion site is located outside of the capsid on the tip of a loop at the 3-fold axis of symmetry, facilitating interaction with the receptors of targeted cells. AAV9-PhP.B has shown to be functional in mouse models. For example, AAV9-PhP.B-based gene therapy has been shown to reduce α-synuclein pathology in a preclinical mouse model (Morabito et al., 2017).

We hypothesized that the addition of PhP.B could also be used to enhance BBB crossing of other serotypes. Zhang et al. (2011) showed that the natural serotypes of AAV2 and AAV5 do not cross the BBB even in neonatal mice (Zhang et al., 2011). AAV5 is the most divergent of the naturally occurring AAVs. After local administration, AAV5 shows widespread transduction of the brain (Samaranch et al., 2017), making this vector a useful candidate to test the limits of PhP.B. This widespread targeting has been shown in a variety of animal models such as mice, rats, nonhuman primates, and minipigs (Emborg et al., 2014; Miniarikova et al., 2017; Evers et al., 2018; Caron et al., 2019). Furthermore, AAV5 has been shown to specifically target astrocytes and specialized neurons such as dopaminergic and motor neurons derived from the human-induced pluripotential stem cells (Martier et al., 2019a). In in addition to neuronal tropism AAV5 could be delivered systemically in a noninvasive manner would be ideal. Therefore, PhP.B was added to AAV5 to explore the enhanced crossing of the BBB.

First, AAV5 and AAV9 were modified by inserting the 7-mer PhP.B, and the vectors were produced in the baculovirus expression system. This system facilitates scaling up production (Kotin and Snyder, 2017). Then, these new capsids were evaluated *in vitro* with respect to the green fluorescent protein (GFP) expression and dose–response quantified by luciferase activity. The capability of capsids to cross the BBB of mice was assessed at a relatively low dose to remain below the saturation threshold. Finally, IStr and IV delivery of AAV5 and AAV9-PhP.B were compared for transduction and transgene expression in the cortex and striatum.

## Materials and Methods

### Vector Design and Production

**FIGURE 1:**
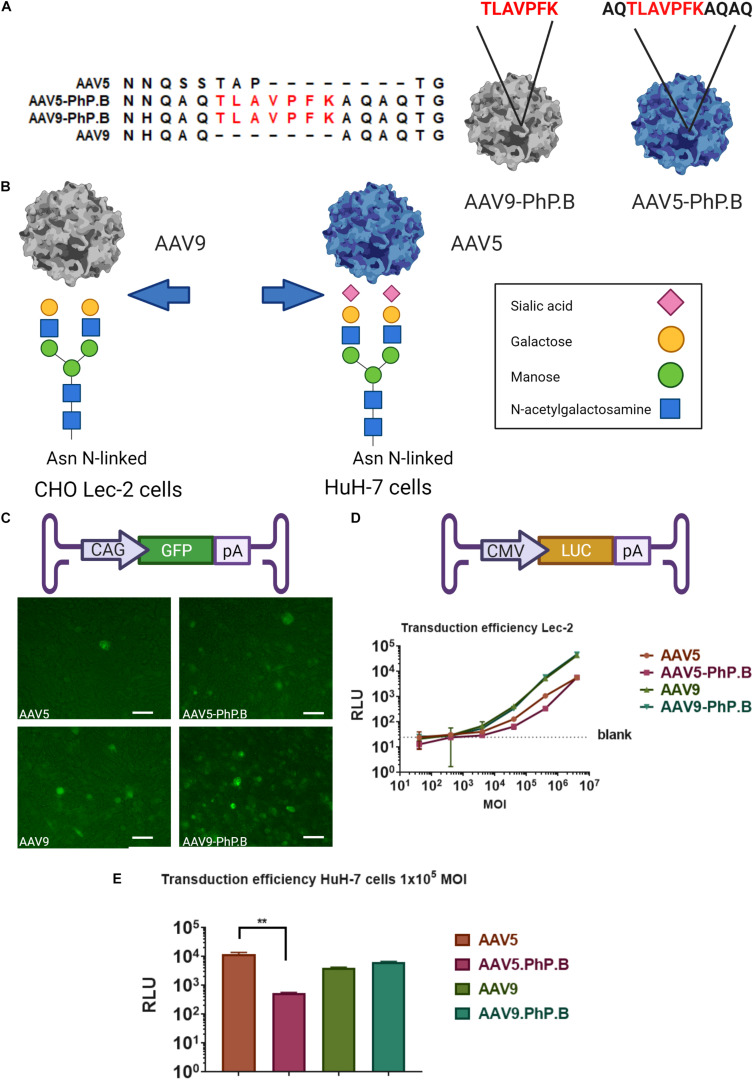
Overview of AAV-PhP.B capsids and *in vitro* testing. **(A)** Alignment of AAV5, AAV5-PhP.B, AAV9, and AAV9-PhP.B at PhP.B insertion. **(B)** Binding by AAV9 of CHO Lec-2 cells due to the modification of the cell surface in glycan receptor exposing galactose, whereas AAV-5 transduces Huh-7 cells through binding to sialic acid. Receptors on the cell surface are depicted in this figure. **(C)** Schematic overview of the expression cassette containing the cytomegalovirus (CMV) early enhancer element and chicken beta-actin promoter (CAG), green fluorescent protein (GFP), and poly(A) tail. CHO Lec-2 cells were inoculated in triplicate with GFP encoding constructs of each capsid. All capsids are capable of inducing GFP expression at 7 days postinoculation. **(D)** Graphical representation of expression cassette used containing CMV-promoter-luciferase gene and poly(A) tail. A dose–response of each capsid in CHO Lec-2 cells measured as relative luminescence (RLU). A total of 2 × 10^4^ cells/well were infected in a serial dilution starting at a multiplicity of infection (MOI) of 4 × 10^6^ cells/well in a 96-well plate. **(E)** Inoculation of HuH-7 cells with AAV vectors. The addition of PhP.B to AAV-5 resulted in a decreased transduction in both cell types. Experiments were performed in triplicate in a 24-well plate, seeding 1 × 10^5^ cells/well for HuH-7 cells. AAV5, orange bar; AAV5-PhP.B, pink bar; AAV9, light green; and AAV9-PhP.B, dark green. Asterisks indicate a statistical significance of *p* ≤ 0.005.

The AAV9-PhP.B transfer plasmid was designed by adding the PhP.B peptide to VP1 between amino acids 588 and 589 of AAV9 adapted for baculovirus production. AAV5-PhP.B was constructed by swapping in the 13-mer loop containing PhP.B, as described in the study of Deverman et al. (2016), and publicly available as sequence KU056473, shown in Figure 1A. Baculovirus was produced by homologous recombination with the transfer plasmids, as described earlier (Urabe et al., 2002; Bosma et al., 2018). Expression cassettes containing either GFP, Luc, or miRNA were generated correspondingly. To generate AAV, *Spodoptera frugiperda* (SF) SF+ cells were triple-infected with baculovirus containing capsid, expression cassette, and the replicon enzyme. At 72 h postinfection, cells were lysed, and the clarified lysate was purified on the ÄKTA explorer (FPLC chromatography system, GE healthcare, United Kingdom) using AVB sepharose (GE healthcare, United Kingdom). The vectors were titrated by SYBR Green for quantitative PCR (qPCR) using a primer pair binding to the promoter region CAG (forward primer: GAG CCG CAG CCA TTG C and reverse primer: CAC AGA TTT GGG ACA AAG GAA GT) or CMV (forward primer: AATGGGCGGTAGGCGTGTA and reverse primer: AGGCGATCTGACGGTTCACTAA) and expressed as genome copies per ml (GC/ml).

### *In vitro* Analysis

Of note, 1 × 10^5^ CHO Lec-2 cells were seeded in a 24-well plate and inoculated and incubated overnight. On the following day, cells were inoculated with 1 × 10^11^ GC/well for a multiplicity of infection (MOI) of 1 × 10^6^ in triplicate for each capsid. GFP expression was visualized 3–7 days postinoculation. CHO Lec-2 cells were seeded at a density of 2.5 × 10^4^ cells/well in duplicate in a black 96-well plate and inoculated in a 10-fold serial dilution starting at MOI of 4 × 10^6^ of each capsid containing a luciferase expression cassette. Plates were incubated at 37°C for approximately 20 h and subsequently analyzed. 1 × 10^5^ HuH-7 cells or 5 × 10^4^ CHO Lec-2 cells were seeded in triplicate in two 24-well plate and inoculated with an MOI of 1 × 10^5^ incubated for 20 h. One plate was used for luciferase analysis and the other for DNA.

Luciferase assay was carried out using the One-Glo Luciferase Assay System (E6120, Promega, Madison, United States) according to the instruction of the manufacturer. DNA was extracted from cell lysate using the AllPrep kit (QIAGEN, Germany) according to the instruction of the manufacturer. Vector DNA was detected by qPCR using SYBR Green primers binding to the CMV promoter (forward primer: AAT GGG CGG TAG GCG TGTA and reverse primer: CAC AGA TTT GGG ACA AAG GAA GT).

### Animal Studies

All animal studies described were approved by the local ethics committee for animal experimentation. Female, young adult C57Bl/6 mice (Janvier Labs, France) were used for all experiments. For IV administration, 8 μl/g body weight of each viral vector preparation was injected into the tail vein. For IStr administration, animals were anesthetized with Hypnorm/Dormicum, and an incision was made in the skin of the head. A small hole was drilled in the skull, and the striatum was stereotactically approached. A 2 μl of vehicle or AAV-miRNA was injected into the striatum in both hemispheres (Anteroposterior = +0.8 mm; mediolateral = ±1.8 mm; Dorsal/Ventral = −3.0 mm) using a 30-Ga needle (BD, Becton, Dickinson and Company, NJ, United States) and attached by tubing to a 10-μl Hamilton syringe at a rate of 0.5 μl/min. After surgery, mice were administered buprenorphine (Temgesic) as pain relief. An overview of injection routes, vectors, and dosage used is given in Table 1.

**TABLE 1:** Overview of injection routes, vectors, and dosage used.

**Study**	**Vectors**	**Injection route**	**Volume (μl)**	**Total dose (GC/mouse)**	**Titer (GC/ml)**
Comparison of capsids at a low dose	AAV5-GFP AAV5-PhP.B-GFP AAV9-GFP AAV9-PhP.B-GFP	IV	200/25 gr	2,5 × 10^11^	1 × 10^12^
AAV9-PhP.B transduction of CNS	AAV9-PhP.B-GFP	IV	200/25 gr	4 × 10^12^	1,6 × 10^13^
AAV5 vs. AAV9-PhP.B	AAV5-miRNA	IV	160/20 gr*	4 × 10^12^	2,7 × 10^13^
	AAV9-miRNA	IStr	4 (2/hemisphere)	1 × 10^11^	2,7 × 10^13^

*Mice were 6 weeks old at the start of the experiment.

### Vector Distribution and GFP Expression

At 4 or 6 weeks postinjection, animals were humanely euthanized, and organs were dissected and frozen at −80°C. For the first experiment, in comparison with capsids at a low dose, the left hemisphere was used for molecular analysis and the other half fixed in 4% paraformaldehyde for histology. For the last experiment, both left and right hemispheres were frozen for the vector distribution analysis.

Organ pieces were pulverized to a powder using the cryoPREP system (Covaris, Woburn, MA, United States). Approximately ± 10-mg powder was used to extract DNA using the DNeasy^®^ 96 Blood and Tissue kit (QIAGEN, Germany). The presence of vector DNA was detected and quantified by qPCR using TaqMan primers and probe binding to CAG promoter (forward primer: GAG CCG CAG CCA TTG C, reverse primer: CAC AGA TTT GGG ACA AAG GAA GT, and probe: ATG GTA ATC GTG CGA GAG GGC GC). Vector DNA genome copies per μg DNA were quantified by interpolating from a standard line prepared from the plasmid of the expression cassette. RNA was isolated from powder using the RNeasy^®^ Plus Mini Kit (74136) from QIAGEN, Germany. Total RNA was reverse-transcribed to cDNA using the Maxima First-Strand kit (R1362, Thermo Fisher, Waltham, MA, United States), RNA expression was quantified by using primers binding to GFP (forward primer: AGC AAA GAC CCC AAC GAG AA, reverse primer: GCG GCG GTC ACG AAC TC, and probe: CGC GAT CAC ATG GTC CTG CT) and GAPDH (TaqMan expression array from Thermo Fisher, Paisley, United Kingdom) as a housekeeping gene for reference. RNA expression was calculated using the ΔCT method normalized to the housekeeping gene.

#### microRNA TaqMan Assay

For the detection of the expressed microRNA, pulverized cortical and striatal tissue was subjected to the Direct-zol kit (R2061, ZYMO Research, Irvine United States) according to the protocol of the manufacturer. RNA was reverse-transcribed into cDNA using the TaqMan^®^ MicroRNA Reverse Transcription Kit (Applied Biosystems, 4366597, Waltham, MA, United States). Diluted samples of each cDNA sample were subjected to TaqMan qPCR using a primer–probe set specific for miRNA and an internal control miRNA (U6).

### Graphs, Statistical Analysis, and Figures

The statistical analysis and graphs were generated using GraphPad, www.graphpad.com [GraphPad Prism version 8.4.2. (679) for Windows, GraphPad Software, San Diego, CA, United States]. To compare groups, a one-way ANOVA was used, followed by the Tukey’s multiple comparisons test. Figures with cartoons were created using biorender.com. Graphical Abstract was made by Sabela DeScience.

### Histology

Of note, 24–48 h after immersion fixation in 4% paraformaldehyde, brains were embedded in 10% gelatin (Difco) in a phosphate-buffered saline (PBS). Embedded tissue was sectioned on a Vibratome. Coronal sections were collected in PBS in series at a thickness of 50 μM. Immunohistochemistry (IHC) was performed on the free-floating sections. Endogenous peroxidase block was performed by incubating sections for 1 h in 1% H_2_O_2_/30% ethanol in PBS. Sections were washed three times with washing buffer (PBS/0.05% Tween). Nonspecific blocking was performed by incubating sections for 1 h in PBS supplemented with 4% bovine serum albumin (BSA) and 5% normal goat serum (NGS). Subsequently, sections were incubated overnight with primary antibody against GFP (Abcam ab290, Cambridge, United Kingdom), diluted 1:1000 in PBS/1% BSA/1.25% NGS/0.5% Tween, and incubated for 1 h with horseradish peroxidase (HRP)-conjugated anti-rabbit before detection with 3,3′-diaminobenzidine (DAB) according to instructions of the manufacturer (Dako EnVision kit K4009, Agilent, Santa Clara, CA, United States). Subsequently, they were dehydrated through ethanol series, xylene, and embedded in Entellan before microscopic analysis.

## Results

### Design and *in vitro* Testing of AAV-PhP.B Capsids

To ensure that the vectors could be adapted to our production system, PhP.B was added to AAVs adapted for the baculovirus expression system. For AAV9, PhP.B was added at the same position, I-588, as described in the study of Deverman et al. (2016).

Earlier attempts to modify AAV5 at the corresponding position I575 were not successful (Khabou et al., 2016). An alignment of AAV5 and AAV9-PhP.B shows sequence similarity outside of the known variable regions, forming a loop at the 3-fold axis of symmetry (Figure 1A). This loop facilitates interaction with the receptors of targeted cells. To add this loop to AAV5, AAV5-PhP.B (i.e., AQTLAVPFKAQAQ) was made by swapping in the flaking regions of AAV9-PhP.B containing the peptide (Figure 1A).

The new capsids were tested for transduction efficacy *in vitro* using Chinese hamster ovary (CHO) Lec-2 cells and HuH-7 cells. Having an easily accessible nonmurine cell line is essential to set up future potency experiments. CHO Lec-2 cells are deficient in monophosphate-SA (CMP-SA) Golgi transporter. Therefore, *N*- and *O*-glycans of these cells only contain galactose residues, as depicted in Figure 1B. CHO Lec-2 cells are suitable for studying AAV9 transduction *in vitro* as this virus uses galactose residues to enter the cell (Bell et al., 2012). HuH-7 cells were used to study AAV5 preferential *in vitro* transduction by sialic acid residues. HuH-7 cells are human differentiated hepatocytes derived from a cellular carcinoma cell line (Figure 1B).

The CHO Lec-2 cells were inoculated with each capsid at an MOI of 1 × 10^6^ GC/cell using GFP as a transgene and monitored over time to assess the ability of capsids to express a transgene. All capsids showed GFP expression 7 days after inoculation (Figure 1C). To verify the potency of AAV-PhP.B, CHO Lec-2 cells were transduced with each capsid in a 10-fold serial dilution, starting with an MOI of 4 × 10^6^ GC/cell. A dose–response was observed for each capsid, showing the potency of the capsids (Figure 1D). No difference was found in the transduction efficiency of AAV9-PhP.B vs. AA9 (Figure 1D). As expected, the potency of AAV5 and AAV5-PhP.B was lower. Surprisingly, in HuH7 cells, the addition of PhP.B to AAV5 resulted in a significant reduction in transgene activity (Figure 1E).

### At a Low Dose, AAV9-PhP.B Can Cross the BBB to Reach the Mouse Brain

**FIGURE 2:**
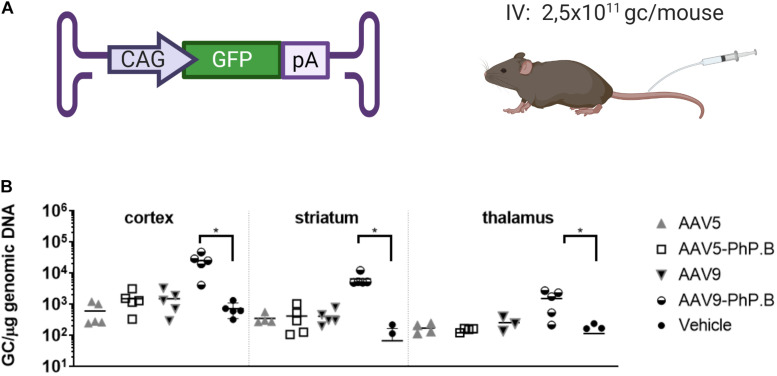
Vector distribution in brain regions of AAV-PhP.B-GFP-administered mice. **(A)** Mice (*n* = 5) were administered 2.5 × 10^11^ GC/mouse by intravenous (IV) injection. The expression cassette used contained the CAG, GFP, and poly(A) tail. **(B)** Tissue was collected 6 weeks after surgery and analyzed for the distribution of the vectors. Significantly more vector DNA was retrieved from the cortex, striatum, and thalamus of AAV-PhP.B-GFP-administered mice when compared to vehicle (PBS). The statistical analysis was performed by a one-way ANOVA followed by the Tukey’s multiple comparisons test, comparing groups with vehicle. Asterisks indicate a statistical significance of *p* ≤ 0.05.

AAV5, AAV5-PhP.B, AAV9, and AAV9-PhP.B were evaluated *in vivo* in C57Bl/6 mice for their capacity to cross the BBB following IV injection and a life period of 6 weeks. Mice were administered a relatively low dose (2.5 × 10^11^ GC/mouse) of each capsid containing GFP as transgene (Figure 2A). Vector distribution was assessed in the cortex, striatum, and thalamus by qPCR. Only AAV9-PhP.B showed significant transduction to all three investigated brain areas (Figure 2B). However, the numbers of vector copies were very low to generate a positive GFP staining on IHC sections. The addition of the PhP.B peptide to AAV5 did not alter the ability of this capsid to cross the BBB (Figure 2B).

### AAV5-PhP.B Shows Increased GFP mRNA Expression in Mice Liver

**FIGURE 3:**
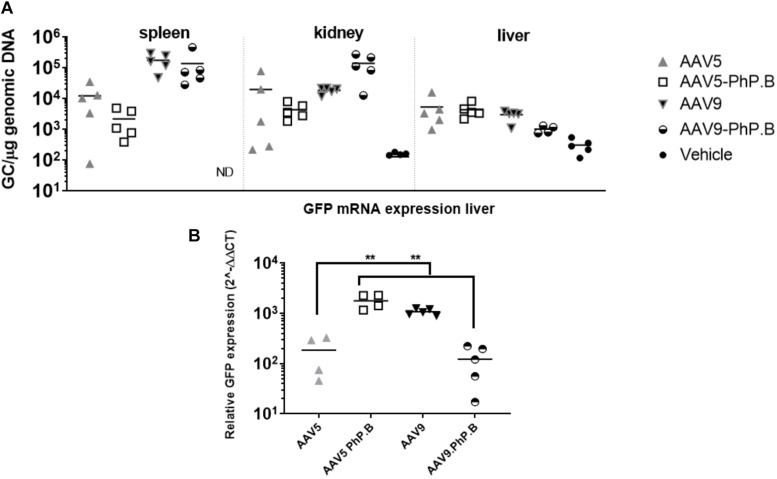
Vector distribution in mice periphery after IV administration of AAV and liver expression. **(A)** Vector distribution in the spleen, kidney, and liver. **(B)** GFP mRNA expression in the liver with elevated levels in AAV5-PhP.B compared with AAV5. Asterisks indicate a statistical significance of *p* ≤ 0.005.

*In vitro* experiments showed a reduction in transduction and luciferase expression in AAV5-PhP.B-transduced HuH-7 cells compared with AAV5 and no difference between AAV9 and AAV9-PhP.B. We also assessed the transduction of peripheral organs *in vivo* and liver GFP mRNA expression in mice. A trend is observed in the higher levels of AAV9 and AAV9-PhP.B vector in the spleen. In the kidney, most of the vector is found in animals administered with AAV9-PhP.B. For the liver, there appears to be an equal distribution with the least amount of vector DNA retrieved from mice administered with AAV9-PhP.B-GFP (Figure 3A). GFP mRNA expression was significantly higher in animals administered with AAV5-PhP.B and AAV9 when compared to mice administered with AAV5 or AAV9-PhP.B (Figure 3B).

### Central Nervous System Transduction of AAV9-PhP.B

**FIGURE 4:**
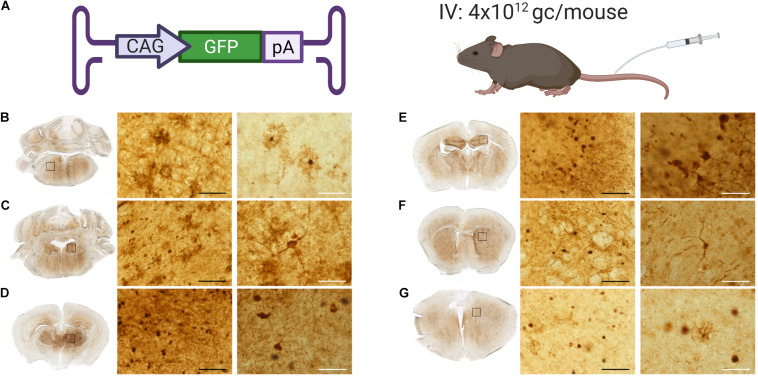
GFP expression throughout the brain of mice administered with AAV9-PhP.B. **(A)** Cartoon depicting construct used, administration route, and dose used of 4 × 10^12^ GC/mouse. Brains were extracted, fixed in 4% paraformaldehyde, embedded in gelatin, and serially cut by using a Vibratome to 50-μm sections. GFP protein staining (shown in brown) in **(B)** the medulla, **(C)** the locus coeruleus, **(D)** the thalamus, **(E)** the hippocampus, **(F)** the striatum, and **(G)** the cortex. Rectangles show the location of the micrograph taken. The black scale bar represents 50 μm, and white bar represents 250 μm.

Genomic copies of AAV were detected and quantified after IV administration of AAV9-PhP.B. However, no GFP expression was observed in brain sections (data not shown) using the relatively low viral dose. To assess the extent of the AAV9-PhP.B-mediated transduction, GFP-transgene-carrying AAV9-PhP.B was administered at a relatively high dose (4 × 10^12^ GC/mouse) to demonstrate the presence of transgene protein within the range of detection (Figure 4A). GFP expression was observed throughout the brain from the medulla to the frontal cortex. Positive neurons and astrocytes were identified by shape and location in the medulla (Figure 4B), the locus coeruleus (Figure 4C), the thalamus (Figure 4D), the hippocampus (Figure 4E), the striatum (Figure 4F), and the cortex (Figure 4G). These results highlight the potential of AAV9-PhP.B to express transgene throughout the CNS.

### Intrastriatal Delivery of AAV5-miRNA Results in High Distribution and Levels of microRNA in Mouse Brain

After establishing that AAV9-PhP.B delivers transgene throughout the mouse brain, we assessed if AAV9-PhP.B could be used for predominantly adult-onset diseases such as HD or Parkinson’s disease. For HD, various huntingtin-gene-expression-lowering strategies are in development (Miniarikova et al., 2018). At the moment, in preclinical studies, IStr administration is the method applied to reach the areas affected in HD. This experiment also aimed to evaluate if AAV9-PhP.B administered by IV could be used as a noninvasive alternative to deliver miRNA in mice.

**FIGURE 5:**
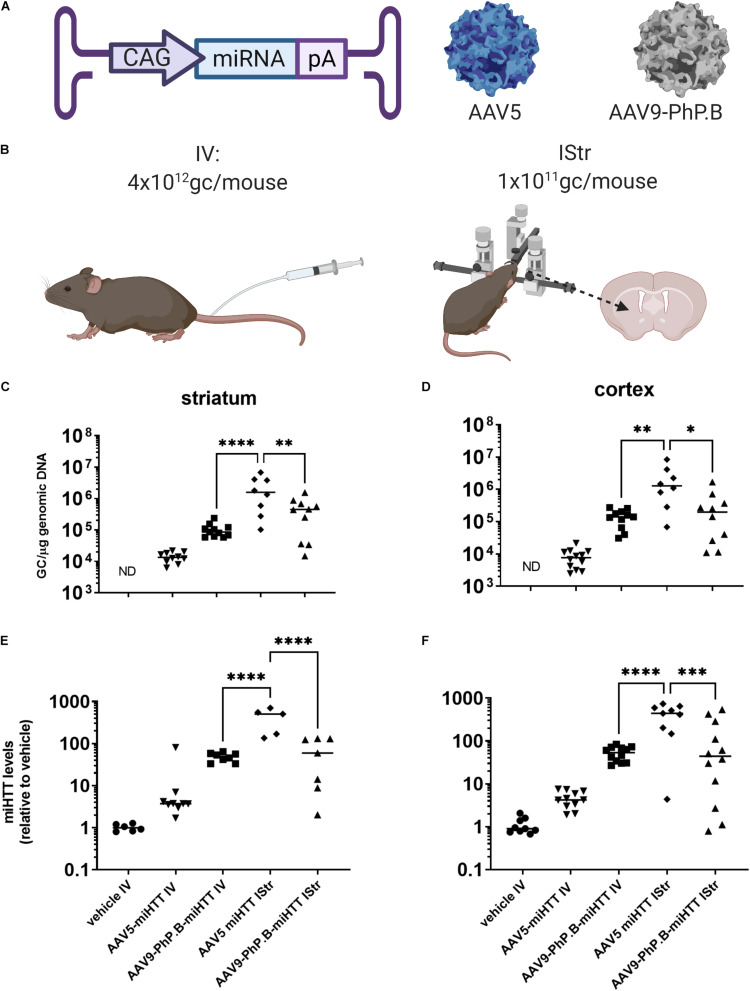
Vector distribution and miRNA expression in mice brain after IV or intrastriatal (IStr) delivery. **(A)** A graphical representation of vectors used in this study. The expression cassette used consisted of CAG-promoter-miRNA and poly(A) tail. **(B)** Administration routes at which mice were either administered intravenously (IV) or IStr. **(C)** Distribution in the striatum. A significantly higher amount of vector DNA was observed in the striatum and cortex of mice that received AAV5 IStr. For AAV5, direct administration results in significantly higher distribution, while for AAV9-PhP.B, similar amounts of the vector were retrieved on average. **(D)** Distribution in the cortex. In the cortex, the same pattern is observed as in the striatum. The IStr administration of AAV5 results in higher amounts of vector DNA than the IV administration of AAV9-PhP.B or AAV5 IV. **(E)** miRNA levels in the striatum and **(F)** miRNA levels in cortex. The vector DNA results translate to miRNA expression levels. In this study, we also observed higher miRNA levels in mice administered AAV5 IStr compared with AAV9-PhP.B IV and AAV5-PhP.B. ND stands for not detected. Statistical analysis was performed by the one-way ANOVA, followed by the Bonferroni multiple comparisons test. Asterisks indicate a statistical significance of ^∗^*p* ≤ 0.05, ^∗∗^*p* < 0.005, ^∗∗∗^*p* ≤ 0.001, and ^****^p < 0.0001.

AAV5 or AAV9-PhP.B containing a miRNA expression cassette (Figure 5A) was produced and administered IStr (1 × 10^11^ GC/mouse) or IV (4 × 10^12^ GC/mouse) at an equal concentration (2.7 × 10^13^ GC/ml), adjusting the volume to the method used (Figure 5B). This is the highest dose that could be produced without the possible formation of aggregates. No significant difference was observed when AAV9-PhP.B was administered IV or IStr. Significantly higher vector genome levels were retrieved from the striatum (Figure 5C) and the cortex (Figure 5D) of mice administered with AAV5-miRNA IStr compared with the other experimental groups. When compared to an IV administration of AAV9-PhP.B, a 22-fold and 15-fold higher vector distribution was achieved in the striatum and cortex, respectively. The levels of miRNA following AAV had a similar pattern as vector DNA found in the striatum (Figure 5E) and the cortex (Figure 5F).

## Discussion

In this study, AAV9-PhP.B and AAV5-PhP.B were produced in the baculovirus expression system and showed to transduce cells *in vitro*. Subsequently, these constructs were evaluated *in vivo*. Although the addition of PhP.B does not enhance BBB crossing of serotype GFP-carrying AAV5, it does increase GFP mRNA expression in the liver. We found that the addition of PhP.B to AAV9 enhances the capability of the vector to cross the BBB, as found in the earlier studies (Deverman et al., 2016; Morabito et al., 2017; Hordeaux et al., 2018), and, when applied at a sufficient dose, GFP expression can be noted throughout the brain from the medulla to the cortex. IStr and IV administration of AAV9-PhP.B mediated similar transduction levels in the cortex and striatum. When we compared IStr administration of AAV5 to IV administration of AAV9, AAV5 IStr was superior in terms of transducing the cortex and striatum.

Overall, we observed lower transduction levels with AAV9 vectors using a similar dose as described in the study first describing PhP.B (Deverman et al., 2016). The difference in the quantification method per laboratory could be a possible explanation for observed differences. In addition, for AAV9, varying results are reported between laboratories with regard to the distribution patterns. For example, in one of the first studies, predominant astrocyte transduction (Foust et al., 2008) was reported while another laboratory observed the transduction of neurons (Gray et al., 2011). Another observation is more variation in the group of animals administered IStr compared with the IV-injected group. Vector spread after the striatal infusion is not homogenous throughout the tissue since this is performed with concentrated vector directly into tissue resulting in local differences. Therefore, more variation is observed between mice. We suspected that transfer of a homogenous dilution in blood from the endothelium to the striatal area occurs in a more homogenous manner and is, therefore, less sensitive to variation between animals.

The addition of PhP.B to AAV5 resulted in increased transgene mRNA in the liver compared with AAV5. Ly6a is constitutively expressed by liver sinusoidal endothelial cells (LSEC) (Luna et al., 2004). The LSEC form a permeable barrier between the bloodstream and hepatocytes (Poisson et al., 2017). In the brain, the interaction between AAV9-PhP.B and Ly6A facilitates the crossing of the BBB. Since we did not note the crossing of the BBB by AAV5-PhP.B, the LSEC may be transduced rather than the hepatocytes underneath.

A recent publication (Powell et al., 2020) showed that AAV9 after infection could mediate expression in different cell types in the brain, depending on the promoter used. Moreover, a six alanine insertion after the VP2 start residue results in a shift from oligodendrocyte to neuronal transduction. The addition of PhP.B to AAV could also have an effect on the specific cells transduced within the tissue. Recently, RNAscope *in situ* hybridization revealed the precise location of the vector DNA and mRNA produced, while identifying specific cells by IHC (Zhao et al., 2020). This method could be applied in the future to investigate and differentiate between level of vector entry and transgene expression in a specific cell. In our case, this would be a method to investigate the liver transduction by AAV5-PhP.B. Since this method is beyond the scope of this study, we did not pursue this further.

The question remains why AAV9 is enhanced by PhP.B to cross the BBB, while AAV5 is not. The mechanism by which AAV crosses the BBB has not been fully understood. AAV9 appears to cross the BBB by transcytosis in an *in vitro* human BBB model by a yet unknown receptor (Merkel et al., 2017). The data in mice indicated that AAV9-PhP.B crosses the BBB by transcytosis of brain microvascular endothelial cells (Matsuzaki et al., 2019). The results of this study with AAV5-PhP.B indicate that PhP.B alone is not enough for an AAV particle to cross the endothelial barrier. Therefore, it appears PhP.B facilitates the transduction of endothelial cells which in turn enables the enhanced trafficking of AAV9 through the endothelium from apical to basolateral side by another co-receptor. For AAV5-PhP.B endocytosis and trandusction of the endothelium might still occur but the astrocytes and neurons underneath remain untransduced.

The addition of different peptides to the corresponding position I587 in AAV2 enhanced the AAV2 ability to transduce the CNS after IV administration (Chen et al., 2009). Therefore, it is theoretically possible to add a peptide to an AAV serotype other than AAV9 and increase BBB crossing. Each of the serotypes uses a different primary receptor. AAV2 binds to heparin sulfate proteoglycan (HSPG), AAV9 to N-linked galactose receptors, and AAV5 to N-linked sialic acid (Vance et al., 2015). However, both AAV2 and AAV9 use laminin receptor (LamR) as a secondary receptor, while AAV5 also binds the platelet-derived growth factor receptor (PDGF-R) (Vance et al., 2015). The interplay between these various receptors appears to play a role in various transduction patterns.

The AAV9-based IV therapy in combination with a PhP.B designed for primate could be a viable option for diseases with multifocal pathologies where different types of cells in the body are affected, such as lysosomal storage diseases (Tardieu et al., 2017). In most cases, ideally, therapy should be applied as early as possible before irreversible neurodegeneration occurs (Massaro et al., 2020). Due to the more permissiveness of the BBB, newborn babies need a lower dose for disease correction, implying a lower risk of the therapy. In diseases where the whole body is genetically affected, the systemic mechanism of AAV9-primate PhP.B might even be an advantage.

For a clinical application, a primate permissive version of PhP.B should be developed. A putative human equivalent of murine Ly6a, the receptor to which PhP.B binds, is Ly6E. The Ly6E protein is highly expressed in human BBB endothelial cells. Furthermore, a peptide sequence within HIV-GP120 is hypothesized as a potential binding motif functioning as a primate permissive PhP.B (Ille et al., 2020). This peptide could be used to develop a human variant of AAV9-PhP.B to be tested in nonhuman primates or *in vitro* systems that mimic the human BBB.

Our results further show that the direct administration of AAV5 represents an attractive strategy for diseases where the deeper brain areas have to be targeted. AAV5 has been studied for localized administration to various CNS targets such as intrathalamic pathway (Samaranch et al., 2017) in the deep cerebellar nuclei (Martier et al., 2019b) and IStr. The IStr administration of AAV5 has been extensively studied in multiple models. Our current transduction results are in line with those of a rodent preclinical HD model in which disease amelioration was achieved (Spronck et al., 2019). The IStr administration of AAV5 has also been studied in nonhuman primates (Samaranch et al., 2017) and minipigs (Evers et al., 2018). A similar transduction pattern is observed across all studied species, with high transduction and transgene expression levels in the striatum and cortex. A clinical study using the IStr administration of AAV5 has been initiated in patients with HD (NCT04120493).

We studied the transduction and miRNA expression in the cortex and striatum following AAV delivery. Irrespective of the delivery method, similar transduction and transgene expression levels are observed in the brain with AAV9-PhP.B, showing that for an AAV9 vector with PhP.B or similar enhancement, there would be no added benefit of an IStr over IV delivery. From the standpoint of a clinical program targeting solely the striatum and the connection areas, it is worth investing in IStr delivery with AAV5 rather than further optimizing AAV9 or another capsid to reach the striatum after IV delivery.

Taken together, our results show that for therapies where high levels of transduction are needed, the direct delivery of AAV5 is a viable option. For treatments in which a wide viral spreading throughout the brain and body is desired, a human-targeting variant of AAV9-PhP.B is an attractive candidate.

## Data Availability Statement

The raw data supporting the conclusions of this article will be made available by the authors, without undue reservation upon request.

## Ethics Statement

The animal study was reviewed and approved by Animal Welfare Body AMC.

## Author Contributions

KP and BB: conceptualization and initial draft. KP, FP, SP, JL, and BB: investigation. SD, PK, GM, and BB: supervision and editing. All authors contributed to the article and approved the submitted version.

## Conflict of Interest

KP, FP, SP, SD, JL, PK, and BB were employees of uniQure at the time the data wad generated. The remaining author declares that the research was conducted in the absence of any commercial or financial relationships that could be construed as a potential conflict of interest.

## Publisher’s Note

All claims expressed in this article are solely those of the authors and do not necessarily represent those of their affiliated organizations, or those of the publisher, the editors and the reviewers. Any product that may be evaluated in this article, or claim that may be made by its manufacturer, is not guaranteed or endorsed by the publisher.
